# A visualization analysis of hotspots and global trends on pelvic floor dysfunction in cervical cancer

**DOI:** 10.1007/s00432-023-05531-2

**Published:** 2024-01-30

**Authors:** Jiawen Wang, Xinhao Wang, Tianming Ma, Yu Lu, Zehao Yan, Jianye Wang, Qiang Hao

**Affiliations:** 1 Department of Urology, Beijing Hospital, National Center of Gerontology, Institute of Geriatric Medicine, Chinese Academy of Medical Sciences, Beijing, 100730 China; 2 Graduate School of Peking Union Medical College, Chinese Academy of Medical Sciences, Beijing, 100005 China; 3 Department of General Surgery, Beijing Hospital, National Center of Gerontology, Institute of Geriatric Medicine, Chinese Academy of Medical Sciences, Beijing, 100730 China; 4 Shanghai First Maternity and Infant Hospital, Tongji University School of Medicine, Shanghai, 200092 China; 5 Department of Urology, Beijing Tiantan Hospital, Capital Medical University, Beijing, 100050 China

**Keywords:** Bibliometry, Pelvic floor dysfunction, Cervical cancer, Sexual dysfunction, Urinary incontinence

## Abstract

**Background/objective:**

Cervical cancer is the major cause of cancer-related mortalities in women globally. It constitutes one of the life-threatening conditions for women in developing countries. The popularization of cervical cancer screening and the improvement of treatment levels has caused the mortality rate of cervical cancer to decrease gradually, but pelvic floor dysfunction before and after cervical cancer treatment has become prominent and attracted more and more attention. Bibliometric analysis has been carried out in this research. The main goal of this research is to provide a comprehensive insight into the knowledge structure and global research hotspots about pelvic floor dysfunction in cervical cancer.

**Methods:**

Literature related to cervical cancer and pelvic floor dysfunction as of May 2023 was searched on the Web of Science Core Collection (WOSCC). The visualization and bibliometric analyses of the number and contents of publications were performed to analyze the temporal trends, spatial distribution, collaborative networks, influential references, keyword co-occurrence, and clustering.

**Results:**

There were 870 publications from 74 countries or regions, with the U.S. publications in a leading position. Since 2020, the number of publications has rapidly increased with the emphasis on the quality of life of cervical cancer patients. Although pelvic floor dysfunction in cervical cancer mainly occurs in developing countries, developed countries have made great contributions to this disease. However, in developing countries such as China and India, the quality of publications needs to be improved. In this field, the studies focused on the sexual dysfunction or urinary incontinence of cervical cancer patients, and the most cited papers discussed the effect of cervical cancer treatment on the sexual activities of females. The frontier keywords were represented by pelvic radiotherapy and risk factors.

**Conclusion:**

This study provides an objective and comprehensive analysis of the literature available on pelvic floor dysfunction in cervical cancer and identifies future trends and current hotspots. It can provide a valuable reference for researchers in this field.

## Introduction

Cervical cancer is one of the most common malignancies of the reproductive system in females and the leading cause of cancer-related mortality in women globally (Rahangdale et al. 2022). According to global epidemiological data, cervical cancer is the fourth most common malignancy that threatens the health of females after breast cancer, lung cancer, and colorectal cancer (Siegel et al. 2023). Human papillomavirus (HPV) has been established as a required element in the formation of cervical cancer (Goodman 2015). The majority of cervical cancer cases can be avoided by vaccinating against the human papillomavirus (HPV) (primary prevention), screening for and treating precancerous lesions caused by HPV infections (secondary prevention), and it can be controlled if treated early (tertiary prevention) (Alfaro et al. 2021). The HPV vaccine has been proven to reduce cervical cancer by 70–90% worldwide (Qu et al. 2023). Although the incidence and mortality of cervical cancer have decreased year by year with the popularization of cervical cancer screening in recent years, it is still a serious health problem and a major public health issue, especially in middle and low-income countries (Boon et al. 2913). It is estimated that there are 604,127 newly-onset cases of cervical cancer every year, 88% of which occur in middle and low-income countries. Besides that, nine out of ten cervical cancer mortalities occur in developing countries (Lintao et al. 2022; Singh et al. 2023).

The treatment procedures employed in the management of cervical cancer have changed greatly. Presently, the treatment methods for cervical cancer mainly include surgery, radiotherapy, chemotherapy, and combination therapy (Moss et al. 2023; Koh et al. 2019; Gupta et al. 2018; Raspagliesi and Bogani 2019). However, regardless of the treatment method, many patients experience varying degrees of pelvic floor dysfunction after treatment (Wenzel et al. 2022; Ziętek-Strobl et al. 2020; Manea et al. 2018). As a complication, pelvic floor dysfunction has greatly limited the therapeutic value of existing treatment methods in patients with cervical cancer (Wit and Horenblas 2014; Nagell et al. 1974).

At present, bibliometric analysis has become a widely used quantitative method to evaluate the quality and influence of academic research results (Brandt et al. 2019). By combining mathematics and statistics and assessing the unique parameters of published literature (such as keyword, author, country, etc.), the status quo, topics, and trends of scientific research can be described quantitatively (Sholklapper et al. 2023). Using this method, a scientific technique is provided for understanding trends in various fields and ranking academic individuals and groups. It also promotes cooperation and exchanges among academic circles, promoting the development and progress of disciplines. In addition, by analyzing bibliometric data and trends, future research directions and development trends can be predicted in the academic field, providing valuable references for decision-makers in academia and related fields (Rossello et al. 2022). However, there are no specific bibliometric studies in the field of pelvic floor dysfunction in individuals with cervical cancer. Therefore, this study aimed to collect and analyze all literature related to pelvic floor dysfunction in cervical cancer as of May 13, 2023, to evaluate the current status of this field and provide directions and suggestions for future research. In this study, pelvic floor dysfunction in cervical cancer was studied systematically to determine the current research focus, trends, and hotspots. The data acquired in this research offers valuable recommendations for future studies, which will help to fully understand the current status of this field. Moreover, the recommendations proposed in this study can serve as a scientific guide and provide support for academic research endeavors in this field.

## Methods

### Data source and search strategy

The literature related to cervical cancer and pelvic floor dysfunction was searched in the Web of Science core database (WOSCC), which covers comprehensive multidisciplinary academic information resources and a vast range of the most influential core academic journals in different research areas. It is the most common bibliometric database (Zhou et al. 2023; Kumar et al. 2023). The download and literature search of all data were completed on May 13, 2023, to avoid the potential bias from frequently updating the database. The search strategy was shown in Table 1.

**Table 1:** Search strategy

#1	“cervical cancer” OR “Uterine Cervical Neoplasms” OR “cervical carcinoma”
#2	incontinence OR “urinary incontinence” OR “urinary incontinence, stress” OR “urinary incontinence, urge” OR “mixed urinary incontinence” OR “pelvic organ prolapse” OR “genital prolapse” OR “uterine prolapse” OR “cervical prolapse” OR “urogenital prolapse” OR “vaginal prolapse” OR “vaginal apex prolapse” OR “vaginal vault prolapse” OR “utero-vaginal prolapse” OR “anterior vaginal wall prolapse” OR “posterior wall prolapse” OR “posterior vaginal wall prolapse” OR rectocele OR proctocele* OR cystocele OR “urinary bladder prolapse” OR enterocele OR “fecal incontinence” OR “anal incontinence” OR “bowel incontinence” OR “sexual dysfunction, physiological” OR “physiological sexual dysfunction*” OR “sexual dysfunction*” OR “sexual disorder*” OR dyspareunia OR “pelvic pain” OR “urinary retention” OR “urinary bladder diseases” OR “urinary bladder, neurogenic” OR “urinary bladder” OR “urinary bladder, overactive” OR “overactive bladder” OR “overactive detrusor” OR “overactive bladder syndrome” OR “urinary bladder, neurogenic” OR “neurogenic bladder” OR “bladder dysfunction” OR “diurnal enuresis” OR “nocturnal enuresis” OR “lower urinary tract symptoms” OR “lower urinary tract dysfunction” OR “lower urinary tract abnormalities” OR “pelvic floor” OR “pelvic floor disorders” OR “pelvic floor disorder*” OR “pelvic floor disease*” OR “pelvic floor dysfunction*”
#3	#1 AND #2

### Data selection

**Fig. 1:**
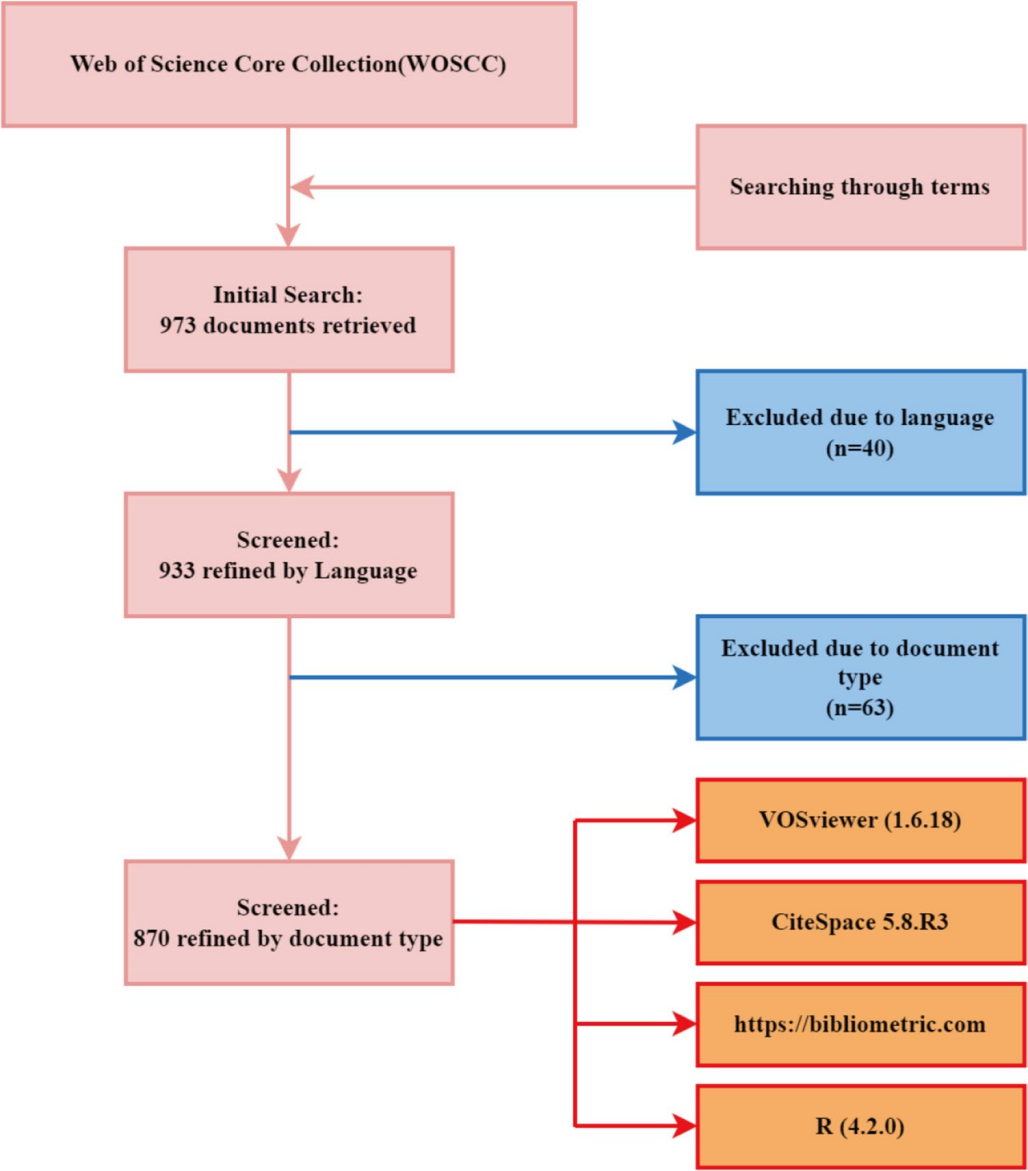
Framework flowchart. The figure reveals detailed selection criteria of publications related to pelvic floor dysfunction of cervical cancer and the steps of bibliometric analysis in the WOSCC database

The publications included in this research were related to cervical cancer and pelvic floor dysfunction, excluding Proceeding Papers, Meeting Abstract, Editorial Material, Book Chapters, Early Access, Letters, Correction and Note, and non-English literature. The two authors independently searched relevant literature in the WOSCC database and downloaded the TXT format (title, article citations, keywords, author information, abstract, countries or regions published and references, etc.). In case of divergence, help from a third researcher was required. The literature search process is illustrated in Fig. 1.

### Bibliometrics and visualization analysis

All valid data were converted to TXT format and then imported into the analysis software. The analysis was performed using CiteSpace (5.8.R3), bibliometric online analysis platforms (http://bibliometric.com/), VOSviewer (1.6.18), and R (4.2.0). Software such as CiteSpace and VOSviewer have become widely used tools in bibliometric analysis (Eck and Waltman 2010; Derviş 2019; Chen 2020). The quality of the included literature was evaluated, and the analysis of the countries, authors, institutions, references, journals, keywords, etc. were performed, with the visualization results generated subsequently. In addition, the analysis of the strongest citation bursts based on keywords was conducted, which could help to understand the trend of the changes in the research hotspots over time (Song et al. 2001).

## Results

### Overall situation of publications

**Fig. 2:**
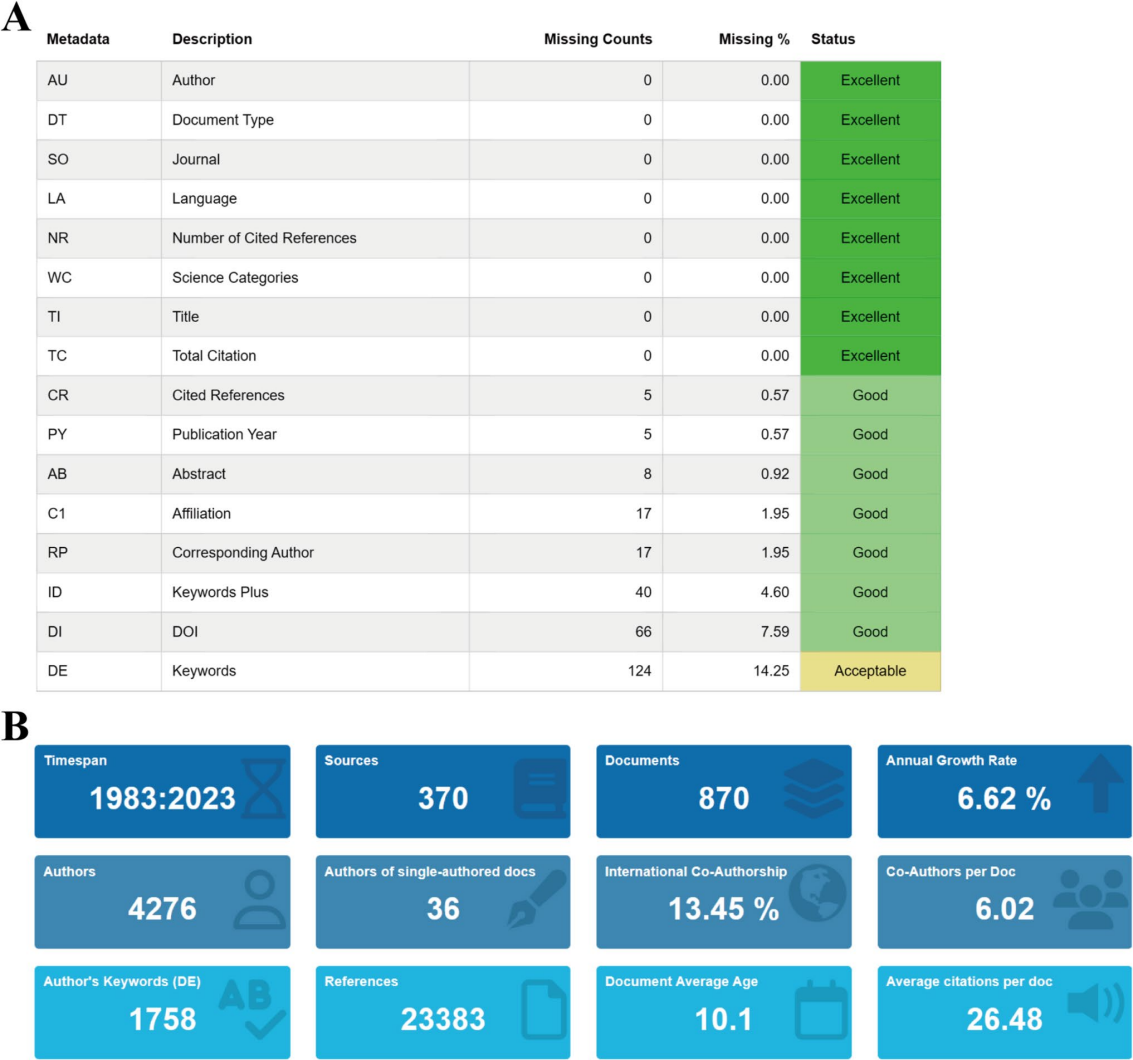
General characteristics of publications. **A** Quality evaluation of publications. **B** Data summary of publications

As of May 13, 2023, overall 870 papers were finally included in the bibliometric analysis by reading the titles, abstracts, and full texts. All literature was published in English from 1983 to 2023. The collected literature was of good quality, and only a small number of them lacked keywords (Fig. 2A). The collected publications were from 370 journals, 4,276 authors, and a total of 23,383 references. Publications from international cooperation accounted for about 13.45%. The overall characteristics of the collected literature are shown in Fig. 2B.

### Number of publications and distribution of publication years

**Fig. 3:**
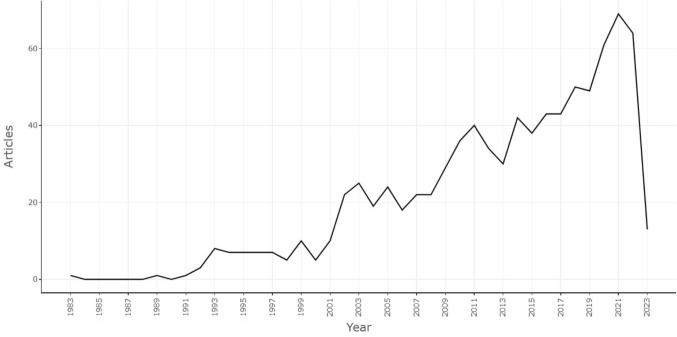
Time trend distribution of publications in the field of cervical cancer and pelvic floor dysfunction

The number of papers published is an important indicator for analyzing the popularity of research fields. In Fig. 3, the chronological distribution of publications by year is shown in the form of a line chart. It was noted that the number of articles related to research on pelvic floor dysfunction in cervical cancer showed an increasing trend year by year, with an annual growth rate of 6.62%. From 1983 to 2001, the number of publications was extremely low and the studies stagnated. However, from 2002 to 2019, the number of publications increased steadily, indicating that the study fields of cervical cancer and pelvic floor dysfunction were attracting more and more attention. A further increase was noted from 2020 to 2022, with more than 60 papers published steadily every year, and it is expected that the number of papers published in 2023 will remain more than 60. This suggests that the study field of pelvic floor dysfunction in individuals with cervical cancer has attracted the attention of researchers.

### Countries and regions

**Fig. 4:**
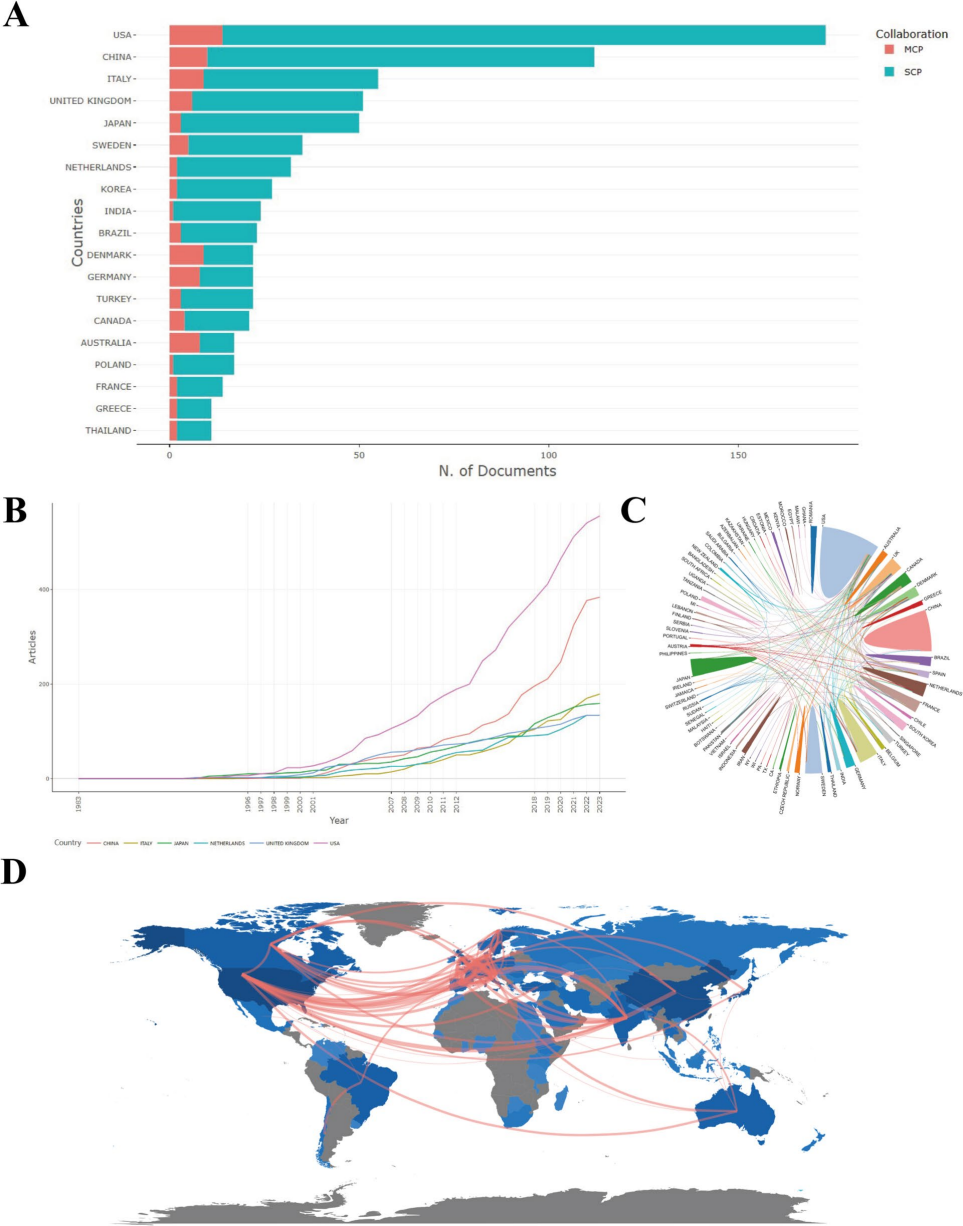
A visualization map of countries. **A** Statistics of the countries with the largest number of publications. (*SCP* single country production; *MCP* multiple countries production). **B** The production trends of countries changed with time. **C** International cooperation between countries that published publications. **D** Country collaboration world map

The literature concerning pelvic floor dysfunction in cervical cancer was published in a total of 74 countries or regions, with the most publications in the United States, followed by China and Italy (Fig. 4A). Figure 4B shows the cumulative number of times the top six countries appeared in the publications over time. The United States has always been far ahead, and China has remained in second place since 2010. International cooperation is a relatively common phenomenon in scientific research, with the most frequent international cooperation with the United States (Fig. 4C, D). Table 2 shows the total number of citations and the average number of citations of the top 10 countries in the field of pelvic floor dysfunction in cervical cancer. Although the number of papers published in China was high, the average number of citations was only 10.7. The papers published in the Netherlands, Sweden, and the UK had a higher number of citations.

**Table 2:** Top ten countries/regions with the most publications and the number of citations

Rank	Country	Documents	Total citations	Average Article citations
1	USA	173	5925	34.2
2	China	112	1195	10.7
3	Italy	55	1366	24.8
4	United Kingdom	51	2460	48.2
5	Japan	50	1112	22.2
6	Sweden	35	1720	49.1
7	Netherlands	32	1573	49.2
8	Korea	27	706	26.1
9	India	24	244	10.2
10	Brazil	23	321	14

### Analysis of universities and institutions

A total of 1331 institutions participated in these articles. Table 3 shows the top ten institutions with the most publications. Among them, 4/10 were from the United States, 2/10 were from Sweden, and 2/10 were from the Netherlands. Karolinska Institute was the institution with the most publications, followed by Peking University, Leiden University, and Memorial Sloan Kettering Cancer Center. The data indicated that universities and institutions in developed countries dominated the research in the field of pelvic floor dysfunction in cervical cancer.

**Table 3:** Top ten universities/institutions with the most publications

Rank	Affiliation	Documents	Country
1	Karolinska Institutet	38	Sweden
2	Peking University	34	China
3	Leiden University	32	Netherlands
4	Memorial Sloan Kettering Cancer Center	32	USA
5	Karolinska University Hospital	24	Sweden
6	University of San Francisco	20	USA
7	Seoul National University	17	Korea
8	University of Utah	16	USA
9	University of Amsterdam	15	Netherlands
10	University of Chicago	15	USA

### Statistical analysis of journals

**Fig. 5:**
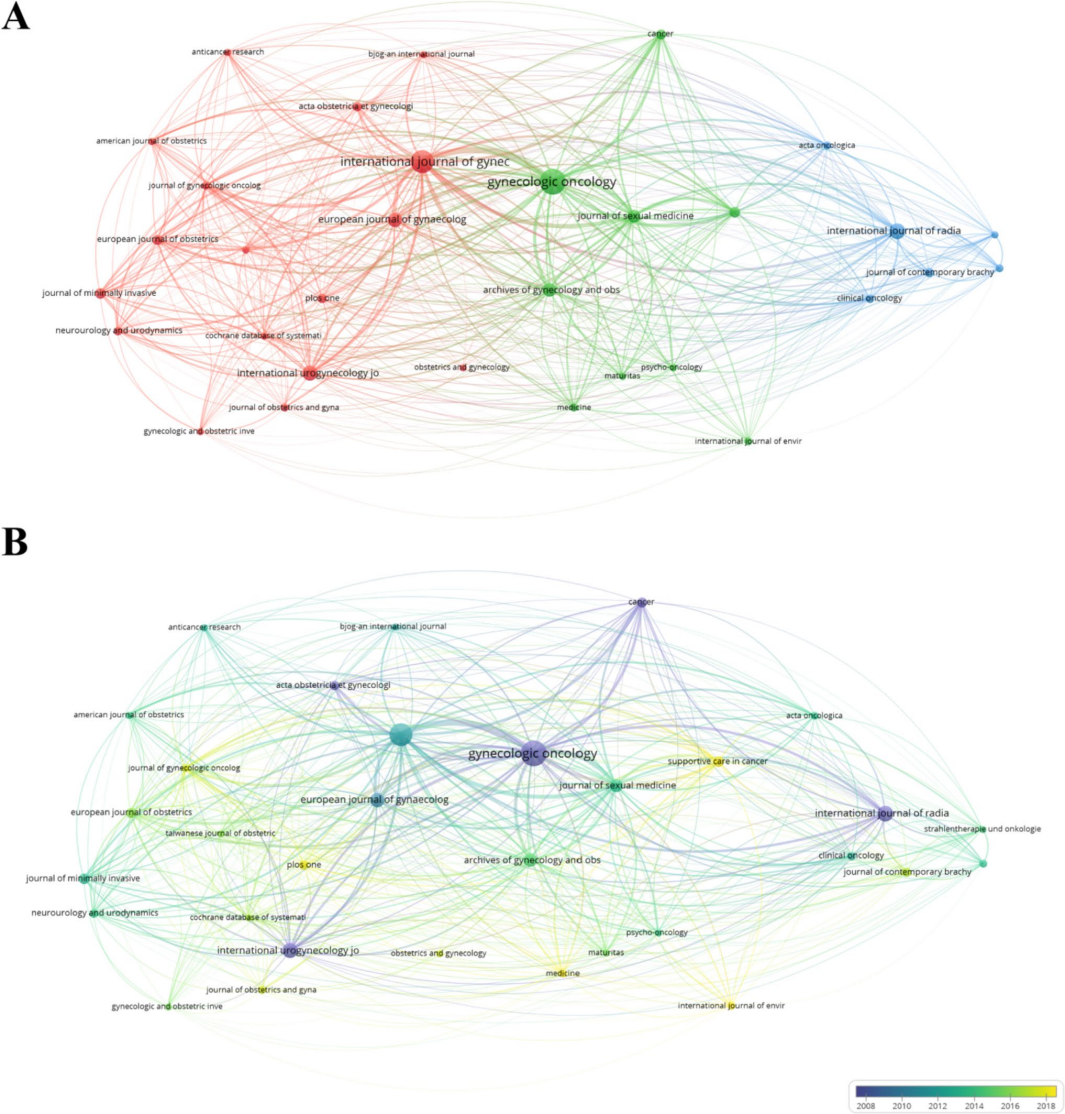
A visualization map of journal bibliography coupling. **A** Network diagram of bibliographic coupling between journals. **B** Bibliographic coupling of journal dynamics and trends

The 870 papers included in this study were published in 370 journals. The top 10 journals and their 2022 impact factors(2022 IF) are listed in Table 4. The number of papers published in Gynecologic Oncology (62, United States) ranked first, with those published in the International Journal of gynecological cancer (49, United States) ranked second, and the International Journal of radiation oncology biology physics publications (24, United Kingdom) ranked third. Gynecologic Oncology had the highest total number of citations. The bibliographic coupling between journals was also visualized (Fig. 5).

**Table 4:** Top ten journals with the largest number of publications

Rank	Source	Documents	Citations	Average article citations	2022 IF
1	Gynecologic Oncology	62	2890	46.6	4.7
2	International Journal of Gynecological Cancer	49	1508	30.8	4.8
3	International Journal of Radiation Oncology Biology Physics	24	1520	63.3	7.0
4	International Urogynecology Journal	22	247	11.2	1.8
5	European Journal of Gynaecological Oncology	19	165	8.7	0.4
6	Archives of Gynecology and Obstetrics	18	195	10.8	2.6
7	Journal of Sexual Medicine	16	585	36.6	3.5
8	Journal of Minimally Invasive Gynecology	11	319	29.0	4.1
9	Supportive Care in Cancer	11	147	13.4	3.1
10	Cancer	10	1128	112.8	6.2

### Statistical analysis of citation frequency of articles

The citation frequency of papers is one of the important indicators to evaluate the quality of research results. Among the 870 selected articles, the top 10 articles were selected according to the citation frequency of the articles as of May 13, 2023. It was noted that the article published by Bergmark in 1999 had the highest citation frequency. In the article, a comparative analysis of 332 women with a history of early cervical cancer and 489 women without cancer showed that women who received treatment for cervical cancer depicted persistent vaginal changes that affected sexual performance and caused considerable distress to patients. Additionally, the articles published by Querleu and Jensen had a citation frequency of 435 and 231 times, ranking them second and third in terms of total citations (Table 5).

**Table 5:** Top ten publications with the highest citation frequency

Rank	Year	Title	First auther	Citations
1	1999	Vaginal changes and sexuality in women with a history of cervical cancer (Bergmark et al. 1999)	Bergmark, K	500
2	2008	Classification of radical hysterectomy (Querleu and Morrow 2008)	Querleu, D	437
3	2010	It's not over when it’s over: long-term symptoms in cancer survivors-a systematic review (Harrington et al. 2010)	Harrington, CB	434
4	2002	Dynamic contrast-enhanced MRI in clinical oncology: Current status and future directions (Padhani 2002)	Padhani, AR	349
5	2004	Posttraumatic stress disorder in female veterans—Association with self-reported health problems and functional impairment (Dobie et al. 2004)	Dobie, DJ	263
6	2010	Radiation dose-volume effects of the urinary bladder (Viswanathan et al. 2010)	Viswanathan, AN	248
7	2007	Second cancers among 104,760 survivors of cervical cancer: evaluation of long-term risk (Chaturvedi et al. 2007)	Chaturvedi, AK	241
8	2003	Longitudinal study of sexual function and vaginal changes after radiotherapy for cervical cancer (Jensen et al. 2004a)	Jensen, PT	231
9	1993	Cancer in kampala, uganda, in 1989–91—changes in incidence in the era of aids (Wabinga et al. 1993)	WABINGA, HR	217
10	2004	Early-stage cervical carcinoma, radical hysterectomy, and sexual function—a longitudinal study (Jensen et al. 2004b)	Jensen, PT	215

As of May 13, 2023

### Statistical analysis of authors

**Fig. 6:**
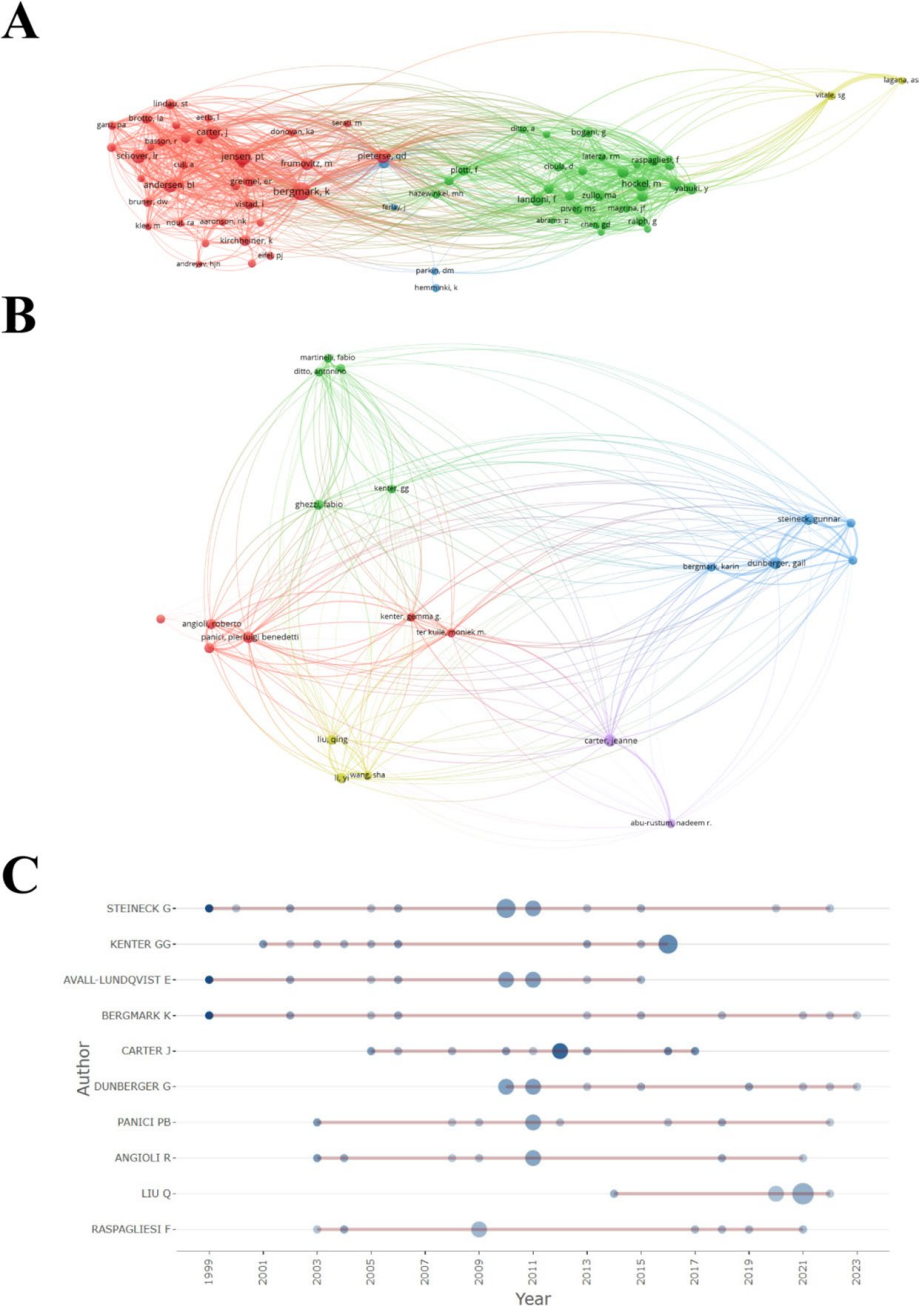
Visualization analysis diagram of the authors. **A** Co-citation analysis among authors (inclusion analysis with a minimum threshold of 30 co-citations). **B** Bibliographic coupling among authors (authors who published more than 5 publications were included in the analysis). **C** Authors' Production over Time

A total of 4276 authors were included in the 870 articles, with the average number of co-authors per paper being 6.02. Only 36 publications had only one author, which indicated that there was a large number of cooperation among the researchers engaged in the study of pelvic floor dysfunction in cervical cancer. The co-citation and Bibliographic coupling between authors were analyzed. The author co-citation analysis identified the core authors in the research areas related to pelvic floor dysfunction in cervical cancer and the association strength between authors (Fig. 6A). The author bibliographic coupling method is an effective method to explore the knowledge structure of a discipline. Hence, it is also a useful supplement to the author co-citation method. Among the authors who have published more than five publications, 21 authors had bibliographic coupling (Fig. 6B). Furthermore, the productivity over the year of the top 10 authors for publishing papers was visualized, with Steineck G indicated as the author with the most publications, followed by Kenter GG and Avall-Lundqvist E.

### Statistical analysis of high-frequency keywords

**Fig. 7:**
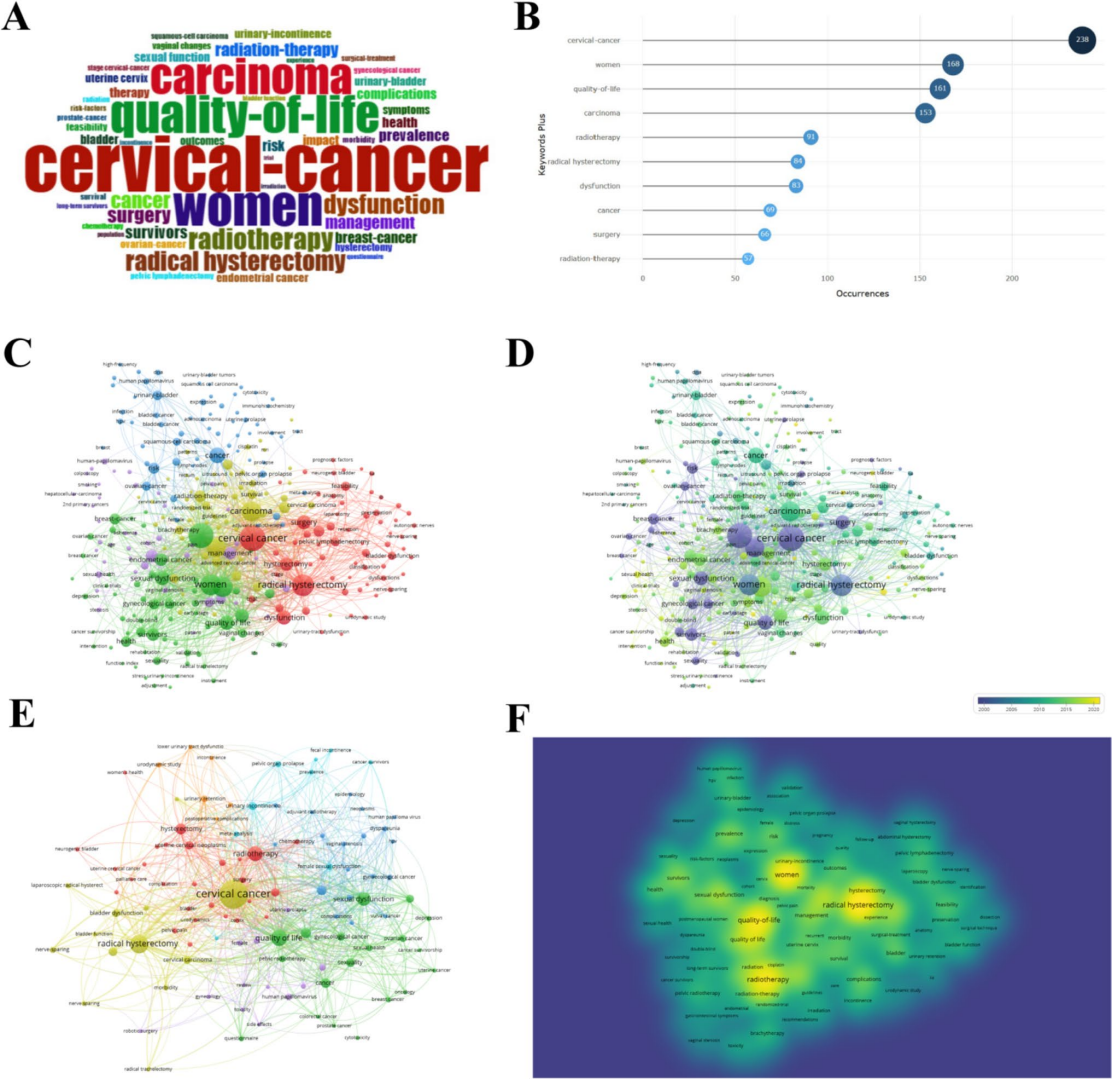
Visualization analysis diagram of keywords. **A** Word cloud based on keywords from KeyWords Plus. **B** Top 10 keywords for the occurrence frequency based on KeyWords Plus. **C** Co-occurrence analysis map of all keywords. **D** Co-occurrence trend over the year of all keywords. **E** Co-occurrence analysis map based on Author keywords. **F** Density map of all keywords after removing tumor-related keywords

The statistical data showed that there were 3481 keywords in 870 articles (including Author keywords and KeyWords Plus). KeyWords Plus is an index word automatically generated according to the title of cited references. It was found that the most frequently occurring keywords in KeyWords Plus were cervical cancer, radical hysterectomy, and quality-of-life, which appeared 261, 185, and 161 times, respectively, and were consistent with the research topic (Fig. 7A, B). Moreover, the analysis of all keywords showed that other keywords with higher frequency were also associated with the quality of life of cervical cancer patients, such as sexual dysfunction, management, dysfunction, women, and complications (Fig. 7C, D). The analysis of author keywords showed that authors preferred using keywords such as quality of life, sexual dysfunction, radical hysterectomy, brachytherapy, and radiotherapy when publishing articles (Fig. 7E). After removing the tumor-related keywords, the research direction of researchers could be identified. Therefore, the keyword density map after removing tumor keywords (Fig. 7F) was drawn. The results showed that sexual dysfunction and urinary incontinence were interesting points for the researchers of pelvic floor dysfunction.

### Analysis of hotspots and trends

**Fig. 8:**
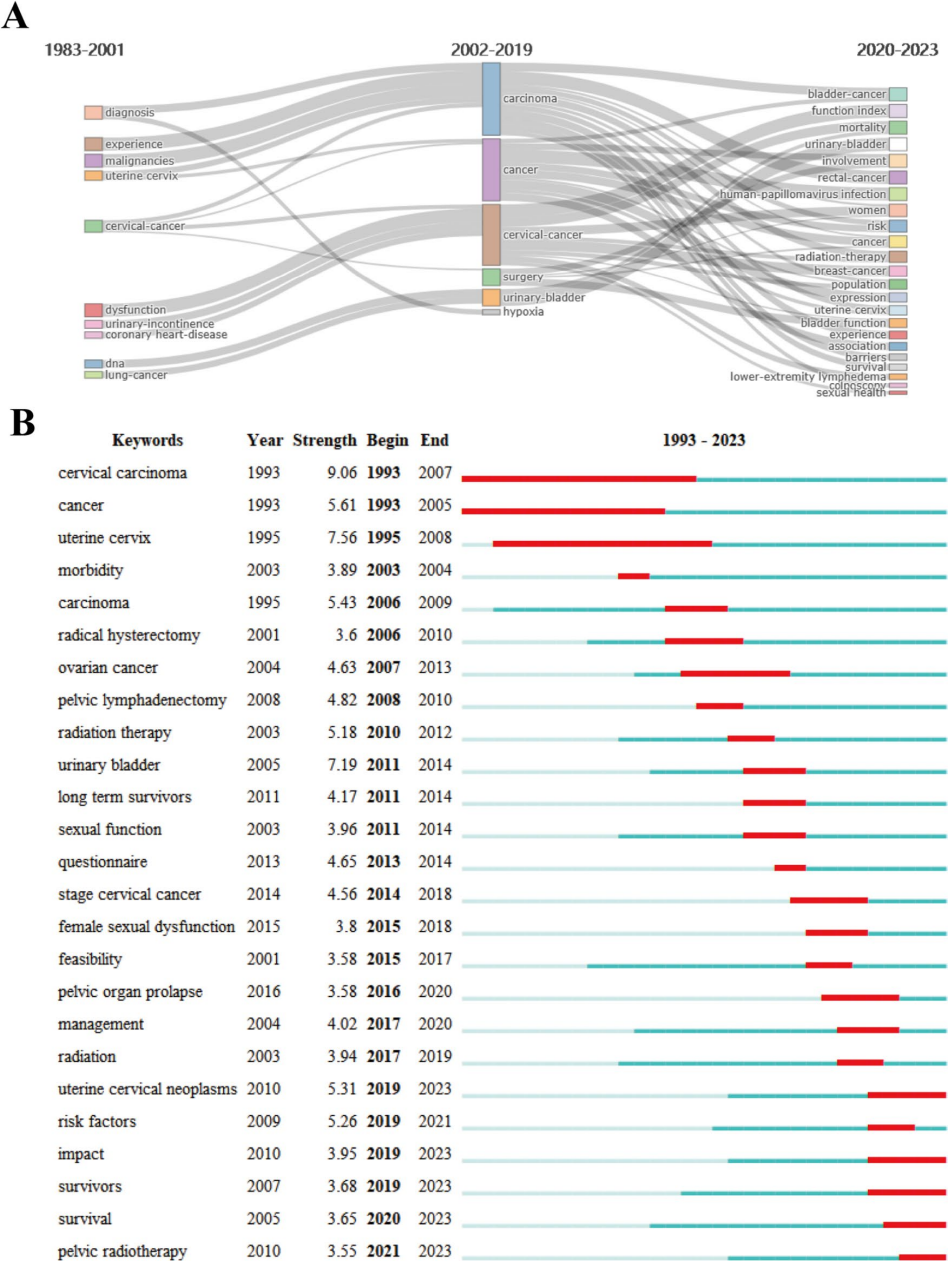
Visualization analysis of hotspots and trends. **A** Thematic evolution. **B** Top 25 keywords with the strongest citation bursts. The blue line represents the time axis, with the red part indicating the start year, end year, and duration of the burst

The analysis of the keywords is helpful to understand research hotspots, frontiers, and trends in this field. According to the number of published papers, all literature was divided into three stages: stagnation period, growth period, and burst period. The period prior to 2001 was considered as a stagnation period. During this period, the number of publications was small, and the keywords were scattered, such as cervical cancer, dysfunction, diagnosis, etc. The keywords in the growth period were mainly tumors, such as carcinoma, cancer, and cervical cancer. Whereas, the keywords in the burst period grew more refined and in-depth, such as radiation therapy, risk, and function index (Fig. 8A). The top 25 keywords with the strongest citation bursts were also analyzed. The time trend/hotspot change showed that cervical carcinoma, cancer, and urine cervix were the hotspots with the longest duration. Additionally, the data indicated that the uterine cervical neoplasms, impact, survivors, survival, pelvic radiotherapy, and risk factors were the hotspots in the recent three years. Furthermore, it was observed that the hotspots, excluding the risk factors, continued to be prominent up until 2023 (Fig. 8B). This suggests that these areas are likely to remain as ongoing research focuses and emerging frontiers in the future.

## Discussion

This study included all publications in the field of cervical cancer and pelvic floor dysfunction from the construction of the WOSCC database to May 2023. The characteristics of publications such as number, countries, institutions, journals, authors, and keywords were analyzed using CiteSpace, VOSviewer, bibliometric online analysis platforms, and R. This study showed that the research in cervical cancer and pelvic floor dysfunction was not a hotspot in the early twenty-first century and prior. However, starting in 2020, there has been a significant increase in research activity in this field, indicating a burst period. The number of published papers has since stabilized at more than 60 (Fig. 3). It is anticipated that studies in the fields of cervical cancer and pelvic floor dysfunction will continue to remain active and experience steady growth in the forthcoming years. This is attributed to the heightened attention from institutions and researchers toward improving the quality of life of cervical cancer patients, especially the functions of the lower urinary tract. This growing focus and support for this field have markedly contributed to the high growth rate in recent years (Hu and Ke 2021; Dogan et al. 2021).

Over the past few decades, the United States has made the greatest academic contribution to the field of pelvic floor dysfunction in cervical cancer, which is reflected in the number of publications. This is evident from the substantial number of publications originating from the United States, accounting for approximately 20%. Furthermore, nearly half of the ten institutions with the most publications are located in the United States. While China ranks as the second largest contributor to the number of publications, only one Chinese institution is included in the top 10 list. Additionally, Italy, Britain, Japan, etc. have also made great contributions to this field. Notably, extensive cooperative research has been carried out among various countries and institutions, highlighting a growing trend towards cooperation in the field of pelvic floor dysfunction in cervical cancer.

The number of citations can represent the quality of papers to a certain extent. Generally, the more citations a published paper has, the higher the quality of the paper and the more original the innovations (Gao et al. 2023; Wang et al. 2022). In this research, the total number of citations and the average number of citations from various countries were analyzed. It was observed that the aforementioned values were higher in the Netherlands, Sweden, and the UK. Although the number of publications was higher in China, the total number of citations and the average number of citations were lower than those in many countries. It is worth noting that although China leads in the number of publications in the field of pelvic floor dysfunction in cervical cancer, the overall quality of the publications needs to be addressed. It is evident that there is a discrepancy between the distribution of published papers and the epidemiological status of cervical cancer. Developing countries, particularly those in sub-Saharan Africa and South Asia, bear a significant burden of cervical cancer-related deaths (88%). However, the top 10 countries with the highest number of published papers predominantly consist of developed countries, with China and India, being the only developing countries (Lancet 2020). This disparity may be attributed to the differing priorities and research focus of these countries. Developing countries focus more on reducing the incidence and mortality of cervical cancer, while developed countries are currently paying more attention to improving the quality of life of patients with cervical cancer. As a result, more studies on pelvic floor dysfunction are conducted in developed countries (Ye et al. 2014). It is important for developing countries to recognize the significance of cervical cancer and actively contribute to research in this area.

In terms of journals, as shown in Table 4, most papers regarding pelvic floor dysfunction in cervical cancer were published in Gynecologic Oncology, followed by the international journal of gynecological cancer and the international journal of radiation oncology biology physics. All of these journals were classified as Q1 in JCR. This means that the quality of published articles in this field is generally high. Papers published in high-IF journals are often cited more, which may provide a theoretical basis for future research and lead the development trend.

The most cited paper is an article published by Bergmark et al. (1999) in The New England Journal of Medicine in 1999. The research dealt with women with cervical cancer, wherein treatment can lead to changes in vaginal anatomy and functions. The researchers recruited 332 women under the age of 80 with a history of early cervical cancer (IB or IIA) who had received treatment in seven gynecological oncology departments in Sweden in 1991 and 1992. The participants were asked to answer questions about vaginal changes and sexual functions in an anonymous questionnaire. The results showed that women who received treatment for cervical cancer depict persistent vaginal changes that can affect the sexual activity and cause considerable distress. In recent years, cervical cancer and sexual activity are still research hotspots in this field. A systematic review encompassing literature from 1966 to 2013 analyzed the changes in sexual function in patients with cervical cancer. The results showed that vaginal dryness, difficulty, short vagina, and sexual dissatisfaction were prominent problems of sexual dysfunction and vaginal changes in cervical cancer (Ye et al. 2014). A survey in Spain showed that almost half of cervical cancer survivors reported sexual dysfunction and impairment of sexual satisfaction (Membrilla-Beltran et al. 2023). This indicates that cervical cancer notably influences the sexual life of patients (Bjelic-Radisic et al. 2012). It is crucial to conduct further studies on the impact of various treatment regimens on sexual functions.

Hotspots refer to scientific topics in a specific research field within a certain period and are one of the key methods of bibliometric analysis (Wu et al. 2021). Keywords usually highlight research ideas, while burst keywords reflect research frontiers and trends (Feng et al. 2023). The analysis shows that radical hysterectomy and radiotherapy are the main research areas of cervical cancer, while sexual dysfunction and urinary incontinence are the research interests of researchers in the field of pelvic floor dysfunction. In terms of the research trends, the studies carried out ten years ago focus on the tumors, while the studies carried out in recent years focus on patient management after treatment to decrease the occurrence of pelvic floor dysfunction and enhance the quality of life. According to burst keywords, the research on the life quality of survivors and the analysis of factors affecting pelvic floor dysfunction are expected to continue being prominent research areas in the future. These areas are likely to continue being frontiers of ongoing research. This indicates that a disease-oriented model has been gradually shifted to a patient-oriented research model. Overall, the research field of pelvic floor dysfunction in cervical cancer appears highly cohesive and focuses on the management of quality of life (Spampinato et al. 2023).

Previous studies have shown that bladder dysfunction is a common complication after radical hysterectomy. The dysfunction arises due to injury or damage inflicted upon the pelvic autonomic nerves that innervate the bladder muscles, urethral sphincter, and pelvic floor fascia (Laterza et al. 2015). It is important to note that radical surgery, radiation therapy, and chemotherapy may lead to pelvic floor dysfunction, including stress incontinence urge incontinence, and dysuria (Vilos et al. 2021; Rogers et al. 2012). Presently, various postoperative interventions have been proposed for the prevention of bladder dysfunction after radical hysterectomy (Aue-Aungkul et al. 2021); however, further studies are needed for pelvic floor dysfunction after radiotherapy and chemotherapy. In terms of treatment methods, doctors often focus on the treatment effect, but ignore the complications after treatment. Further exploration is essential to understand the impact of different treatment methods on pelvic floor dysfunction in patients with cervical cancer. It is necessary for doctors to be aware of and consider the advantages and disadvantages associated with these therapeutic measures during the diagnosis and treatment of cervical cancer.

This research has certain limitations. Firstly, the monographs and reviews written in English and recorded in the WOSCC were included, with the research in other databases and non-English publications excluded. Although this study method may have overlooked some valuable studies, given that WOSCC is the most commonly used scientometric analysis database and covers the vast majority of studies, it has no material impact on the overall trend. Secondly, due to the delay in citations, high-quality studies published recently may not receive the attention they deserve, so they need to be updated accordingly in subsequent studies. Nonetheless, this research will greatly help researchers understand trends, developments, hotspots, and frontiers in the fields of cervical cancer and pelvic floor dysfunction and identify the areas that need to be further studied.

## Conclusions

In conclusion, this bibliographic analysis had the following outcomes:

(1) The number of publications on pelvic floor dysfunction in cervical cancer has increased significantly, and this field is receiving more and more attention; (2) Countries including China, the United States, the United Kingdom and Italy have made great contributions to this research field and researchers have established strong cooperation; (3) Developed countries have made more significant contributions to this field, while developing countries such as China and India need to improve the quality of published publications; (4) The disease-oriented models have gradually shifted to patient-oriented research model, and the life quality of the affected individuals is getting more and more attention; (5) The research topic on sexual life after cervical cancer treatment is still a hot topic, which needs to be further explored as an independent topic; (6) Further exploration is warranted to investigate the impact of different treatment methods on pelvic floor dysfunction in individuals with cervical cancer. It is essential for healthcare providers to be aware of and consider the advantages and disadvantages associated with these treatment approaches during the diagnosis and treatment of cervical cancer; (7) Intervention measures to lower the complications of pelvic floor dysfunction in cervical cancer are a potential research direction.

## Data Availability

The data is completely public and sourced from the Web of Science.
